# Research on Utilizable Calcium from Calcium Carbide Slag with Different Extractors and Its Effect on CO_2_ Mineralization

**DOI:** 10.3390/ma17051068

**Published:** 2024-02-26

**Authors:** Yantao Ma, Xiang Zhang, Zhengyu Du, Haobo Hou, Yiguang Zheng

**Affiliations:** 1 China Power Engineering Consulting Group, Central Southern China Electric Power Design Institute Co., Ltd., Wuhan 430071, China; mayantao@csepdi.com (Y.M.); zhangxiang@csepdi.com (X.Z.); duzhengyu@csepdi.com (Z.D.); 2 School of Resource and Environmental Sciences, Wuhan University, Wuhan 430079, China; 3 Institute of Resources and Environmental Technology, Wuhan University (Zhaoqing), Zhaoqing 526200, China

**Keywords:** indirect carbonation, utilizable calcium, calcium carbide slag, ammonium extract, acid extract, vaterite, calcite

## Abstract

With the increasing accumulation of alkaline industrial solid waste, the mineralization of CO_2_ using alkaline industrial solid waste has broad application prospects. Carbide slag is highly alkaline and contains a large amount of calcium elements, making it an excellent material for CO_2_ mineralization. Our idea was to acquire qualified products and fast kinetics by integrating carbide slag utilization and carbon reduction. The reaction route was divided into two steps: calcium extraction and carbonization. In order to achieve efficient extraction of utilizable calcium, we selected NH_4_Ac as the extraction agent, which has the advantage of buffer protection and environmental friendliness due to being an acetate radical. The extraction efficiency of utilizable calcium exceeded 90% under the conditions of L/S 20:1 and NH_4_^+^/Ca^2+^ 2:1. In the carbonization process, the crystal forms of CaCO_3_ synthesized by direct carbonation, acid extraction, and ammonium salt were characterized. The formation mechanism of vaterite in ammonium solution and the influence of impurities (Al^3+^, Mg^2+^) on the crystal transformation were revealed. This study provides technical support for using alkaline industrial waste to prepare high-purity vaterite. Therefore, alkaline industrial waste can be efficiently and sustainably utilized through CO_2_ mineralization.

## 1. Introduction

The phenomenon of global warming is attributed to anthropogenic emissions of greenhouse gases [1]. The significance of carbon dioxide as the primary greenhouse gas necessitates a focus on reducing its emissions in order to achieve carbon neutrality [2,3]. CO_2_ mineralization is a process by which carbon dioxide reacts with minerals to form solid carbonates [4]. CO_2_ mineralization technology is the process simulating the natural weathering reaction in which Ca or Mg silicates are transformed into carbonates via reaction with gaseous or aqueous carbon dioxide [5]. CO_2_ mineralization technology is considered an effective carbon reduction method [6]. Different mine waste materials have been studied in efforts to understand their potential of mineral carbonation and the effectiveness of different carbonation methods [7]. The process of CO_2_ mineralization can be conducted utilizing either natural minerals or industrial alkaline solid wastes [8,9,10]. There has been pioneering research that has established the chemical and physical derivatives and modalities that drive and control mineralization on several different substrates [11].

The mineralization of CO_2_ using alkaline industrial solid waste has been extensively studied due to the intrinsic alkalinity and Ca/Mg-rich advantages of such waste [12,13,14]. Calcium carbide slag (CS) is a solid waste produced by chlor-alkali industry, which contains a number of alkaline substances [15,16]. Wang et al. pointed out that the development of calcium carbide slag treatment technology is limited due to the high treatment cost and low utilization rate [16]. Calcium-cycling technology is a common way for calcium carbide slag to absorb CO_2_ [17]. However, its application is limited by the deactivation of calcium-based adsorbents [18,19]. Mineralized slag can also be used in building materials, for instance in the preparation of steel slag–gypsum carbide building materials [20]. CO_2_ mineralization studies have also been conducted on other alkaline industrial solid wastes, such as high-calcium fly ash [21,22,23,24] and construction waste [25,26]. These alkaline industrial solid wastes exhibit excellent CO_2_ mineralization capacity. In addition, they can produce usable by-products during the mineralization of CO_2_, which improves the economic profit of the process.

The process of CO_2_ mineralization can be categorized into direct and indirect modes based on the mechanism of mineralization [27]. The indirect mineralization process has attracted much attention due to its mild conditions and high mineralization efficiency [28,29]. Li et al. used a bubble column reactor for direct aqueous mineral carbonation of carbide slag [30]. Aqueous mineral carbonation is limited by the low purity of the product, resulting in low economic benefits [31,32]. Indirect mineralization uses acid or salt solutions to selectively leach Ca^2+^ [28,29]. Li et al. extracted Ca^2+^ from phosphogypsum with acid [33]. In the indirect mineralization of acid extraction, there is low economic feasibility due to the fact that acid is unrecyclable and corrosive [34]. In order to improve the efficiency of mineral extraction and achieve the recovery and reuse of extractants, researchers have focused extensive attention on the process of ammonium salt extraction [10,13,35]. Eloneva et al. selectively extracted calcium from steel slag through ammonium salt solution to prepare CaCO_3_ [36]. NH_4_Cl is an extractant commonly used in the process of leaching Ca^2+^ from calcium carbide slag [37]. To avoid problems caused by Cl^−^, other ammonium salt extractants were investigated in [38]. Li et al. used (NH_4_)_2_SO_4_ to transfer calcium elements to solid phase calcium sulfate and used dropwise carbonization to produce CaCO_3_ [39]. These CO_2_ mineralization techniques present difficulties in ensuring product purity, and more effective extractant solutions need to be studied.

CaCO_3_ with spherical particle morphology(vaterite) has high economic value because of its large specific surface area, dispersibility, and low specific gravity [40,41]. Among the polymorph modifications of CaCO_3_, the metastable vaterite is the most practical inorganic filler and reinforcing agent in the pharmaceutical industry, the paper industry, and the rubber industry [42,43,44]. Vaterite is usually prepared by coprecipitation and carbonation in industry [45]. However, both methods have the problem that vaterite with good crystal form and particle size can only be prepared in aqueous solution with low Ca^2+^ concentration [45]. Increased Ca^2+^ concentration leads to a transformation of the crystalline form, with the coexistence of vaterite and calcite [46]. The extraction of Ca^2+^ from alkaline industrial solid waste by ammonium salt solution to mineralize CO_2_ is considered to be a promising preparation of vaterite [47,48].

In this paper, calcium that can be mineralized by CO_2_ in calcium carbide slag is defined as utilizable calcium. Instead of the traditional NH_4_Cl extractant, NH_4_Ac solution with an organic acid group was selected as the representative of the ammonium extraction process, as it reduces equipment corrosion and related problems. Furthermore, the buffering effect of acetate at the end of the mineralization reaction can delay the further reaction of CaCO_3_. In contrast to similar recent studies on CO_2_ mineralization using ammonium salt, which focus on a single CaCO_3_ synthesis pathway and ignore the influence of impurities on the crystal form of CaCO_3_ [37,49], this work studied the process conditions of ammonium acetate extraction of calcium carbide slag, and the effect of the acid extraction and ammonium salt extraction processes on the selective dissolution of Ca^2+^ was compared. High selective dissolution of utilizable calcium from industrial solid waste was achieved. This research unified the process of calcium extraction with CO_2_ mineralization. Three kinds of CaCO_3_ were synthesized through different mineralization pathways, and these were characterized to study the effect of different methods on mineralization. The mechanism of CaCO_3_ crystal formation during carbonation is here discussed. Furthermore, the influence of the impurity elements (Al^3+^, Mg^2+^) in the carbonization system was investigated. This study provides technical support and suggestions for program selection for using other alkaline industrial solid waste to prepare high purity vaterite.

## 2. Experimental Section

### 2.1. Equipment and Materials

**Figure 1:**
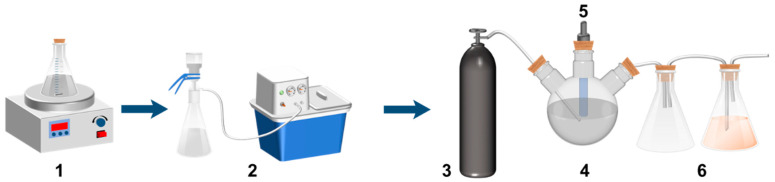
The experimental set-up. (**1**)Utilizable calcium extraction device. (**2**) Vacuum filter extractor. (**3**) CO_2_ gas cylinder. (**4**) Mineralization equipment. (**5**) pH probes. (**6**) Tail gas treatment.

In conical and three-necked flasks, utilizable calcium extraction from carbide slag and carbon dioxide mineralization experiments were conducted using a system consisting of a calcium extraction device, a solid–liquid separation device, and a carbon dioxide mineralization device. The calcium extraction device comprised a conical flask, a magnetic stirrer, and a magnetic bar, while the solid–liquid separation device used a sand core funnel for vacuum filtration. The carbon dioxide mineralization device included a carbon dioxide cylinder (40 L, 99.9%) for pressurized gas supply, a pH probe (range 0–14, accuracy: 0.25%FS), and a tail gas treatment device. The experimental setup is shown in Figure 1.

The carbide slag used in this experiment was an industrial waste slag produced by the PVC industry [50] and originating from Yichang, Hubei. Two representative extractants were selected to extract the utilizable calcium from the carbide slag. In the ammonium extraction process, ammonium acetate (NH_4_Ac) was selected as the representative ammonium salt extractant, on the one hand to explore the influence of ammonium root extractant and the traditional acid extraction process, and on the other hand to explore the influence of ammonium salt organic root on the reaction system. In the traditional acid extraction process, hydrochloric acid(HCl) was used as the extractant for the acid extraction process [51]. In the carbonation stage, the gas used was pure carbon dioxide pressurized from a carbon dioxide cylinder. The carbide slag was dried in a blast-drying oven for 12 h prior to the experiment to remove moisture and weighed.

Based on the L/S and NH_4_^+^/Ca in the experimental research, a specific volume and concentration of extractant solution was prepared and reacted with the carbide slag in a conical flask in a closed manner. After the reaction was completed, the available calcium component was transferred from the carbide slag solid to the liquid phase, and the insoluble impurities were removed by vacuum filtration. The obtained filtrate was placed in a three-necked flask, and the gas inlet pipe, pH probe, and tail gas treatment device were installed. The reaction conditions were set, and carbon dioxide mineralization was initiated. After the reaction proceeded to the end point, it was stopped. The final product of calcium carbonate was separated using solid–liquid separation by vacuum filtration, rinsed with anhydrous ethanol, and dried in a blast-drying oven.

### 2.2. Measurement Methods

#### 2.2.1. Carbide Slag Analysis

The chemical and mineral composition of the carbide slag was analyzed using X-ray fluorescence spectroscopy (XRF) and X-ray diffraction spectroscopy (XRD) analysis methods. Prior to XRF (EDX 4500, Kunshan, China) measurement, the carbide slag sample underwent ignition loss treatment. The main chemical reactions that occurred are shown in Equations (1)–(3). XRD (Bruker D8-Advance, Billerica, MA, USA) analysis was used to detect the mineral composition and crystal structure of carbide slag, with a scanning range of 5–80° and a scanning speed of 5°/min. The carbide slag sample was sieved with different mesh sizes, and the screened samples were analyzed by XRD to determine the differences in the chemical composition of carbide slag samples across different particle size ranges. The compound form and relative content of calcium elements in carbide slag were determined using a thermogravimetric analyzer. The sample was heated from 50 °C to 110 °C at a rate of 10 °C/min under a nitrogen atmosphere, kept for 10 min, and then heated to 1000 °C at a rate of 10 °C/min. The weight loss was used to determine the content of calcium hydroxide, calcium sulfate, and calcium carbonate in different temperature ranges [52]. Fourier transform infrared spectroscopy (FTIR) was used to observe the chemical functional groups of carbide slag to support the results of thermogravimetric analysis. Scanning electron microscopy (SEM) was used to observe the microstructure of carbide slag, and energy dispersive spectrometer (EDS) was used to analyze the surface element composition of the microstructure. The concentration of calcium elements was measured using atomic absorption spectrometry, and the concentration of impurity metal elements was measured using inductively coupled plasma atomic emission spectrometry (ICP-OES).

(1) $$Ca{\left(OH\right)}_{2\left(s\right)}\to {CaO}_{\left(s\right)}+H_{2}O_{\left(g\right)}$$

(2) $${CaCO}_{3\left(s\right)}\to {CaO}_{\left(s\right)}+{CO}_{2\left(g\right)}$$

(3) $${CaSO}_{4}\cdot {2H}_{2}O_{\left(S\right)}\to {CaSO}_{4\left(s\right)}+{2H}_{2}O_{\left(g\right)}$$

#### 2.2.2. Reactions of Carbide Slag Leaching

The calcium extracted from carbide slag is referred to as utilizable calcium. The chemical reaction involved in the stage of utilizable calcium extraction are shown in Equation (4). Calcium hydroxide (Ca(OH)_2_) is slightly soluble in water. During the leaching process, ammonia (NH^4+^) reacts with Ca(OH)_2_, primarily to transfer calcium to the soluble calcium salt phase. This reaction also generates ammonia gas, which dissolves in water to form ammonium hydroxide solution, providing an alkaline environment for subsequent CO_2_ mineralization. In the acid leaching process, hydrogen ions (H^+^) participate in reactions with most inorganic salts in carbide slag, ultimately generating a neutral calcium salt solution and H_2_O.

(4) $${2NH}_{4\left(aq\right)}^{+}+Ca{\left(OH\right)}_{2\left(s\right)}\to {{Ca}^{2+}}_{\left(aq\right)}+{2NH}_{3\left(g\right)}+2H_{2}O$$

#### 2.2.3. Reactions of Utilizable Calcium Mineralization

Equation (5) lists the main reaction involved in the CO_2_ mineralization reaction stage. When utilizable calcium exists in the liquid phase in the form of ions, the solution requires an alkaline environment to capture CO_2_ and generate calcium carbonate. In the ammonium ion extraction process of utilizable calcium, the reaction product ammonium hydroxide can capture CO_2_ without pH adjustment. This process has the advantage of ammonium salt recycling, as shown in equation. In the acid extraction process, alkaline substances need to be added to the reaction system to capture CO_2_ and prepare lightweight calcium carbonate. After CO_2_ is introduced into the reaction system containing alkaline and utilizable calcium, CO_2_ first transfers from the gas phase to the liquid phase, reacts with water to generate carbonic acid, reacts with ammonium hydroxide to generate ammonium carbonate, and then combines with calcium ions in the liquid phase to form solid calcium carbonate. The CO_2_ gas–liquid mass transfer process is the main rate-limiting step of this reaction stage, while the other reactions are fast.

(5) $${CO}_{2\left(g\right)}+{{Ca}^{2+}}_{\left(aq\right)}+H_{2}O+{2NH}_{3\left(g\right)}\to {CaCO}_{3\left(s\right)}{+2NH}_{4\left(aq\right)}^{+}$$

#### 2.2.4. Leaching Efficiency and CaCO_3_ Analysis

The extraction efficiency of calcium from carbide slag was determined by the Formula (6). The carbonate ion concentration in the solution was determined by inductively coupled plasma (ICP) to calculate the calcium ion content Nca in the original carbide slag using microwave acid dissolution. The calcium ion concentration C_ca2+_ in the solution was determined by atomic absorption spectroscopy to estimate the number of calcium ions available for extraction. The extraction efficiency of calcium was calculated by (7), based on the actual carbon dioxide produced and the theoretical carbon dioxide produced. The crystal structure, microstructure, and grain size of the carbonated product were characterized by various methods. The influence of different preparation methods on the product properties were also investigated.

(6) $$\tau =\frac{C_{{Ca}^{2+}}}{N_{ca}}\times 100\%$$

(7) $$\gamma =\frac{{{CaCO}_{3}}_{reacted}}{{{CaCO}_{3}}_{theoretical}}\times 100\%$$

## 3. Results and Discussion

### 3.1. Physico-Chemical Properties of the Carbide Slag

Following ignition loss treatment, the chemical composition of carbide slag was analyzed using X-ray fluorescence (XRF), and the results are presented in Table 1. The primary constituent of the slag was calcium salt, while the impurities included aluminum salt, magnesium salt, and silicon dioxide.

**Figure 2:**
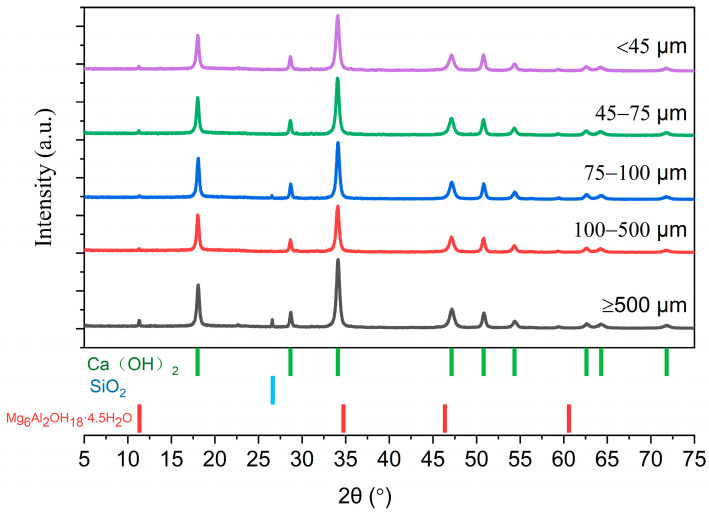
XRD analysis of calcium carbide slag in different particle size ranges.

X-ray diffraction (XRD) analysis was conducted on carbide slag samples with varying particle sizes to investigate their mineral composition. The results are presented in Figure 2. The XRD analysis revealed that Ca(OH)_2_ was the primary crystal structure of the carbide slag, and was the main phase in different particle size ranges. The XRD analysis results of calcium carbide slag are in agreement with the literature [37]. In carbide slag agglomerates larger than 500 μm, impurity peaks of silicon dioxide and magnesium–aluminum complex salt were observed. The carbide slag samples used in subsequent experiments were physically purified using a sieve to remove larger impurities and agglomerates.

**Figure 3:**
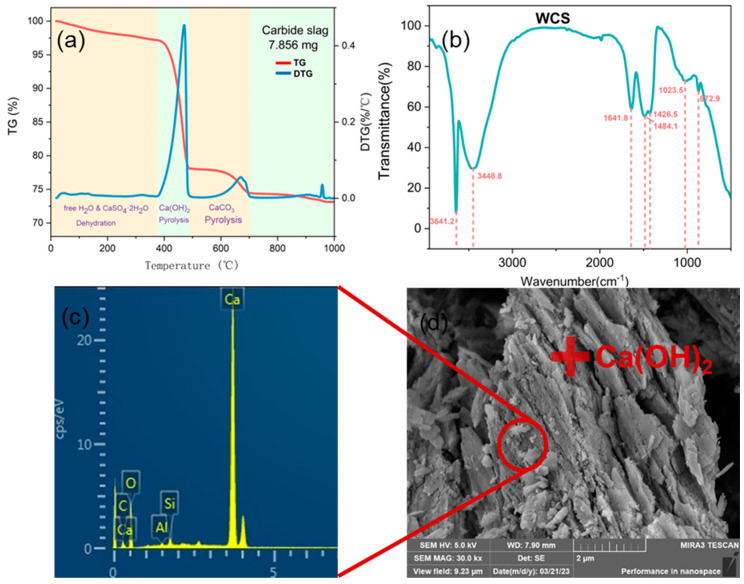
(**a**) Thermogravimetric, (**b**) FTIR, (**c**) energy spectrum, and (**d**) electron microscopy analysis curves of the Carbide slag.

Carbide slag contains various calcium salts. To investigate the compound form of calcium elements in the carbide slag, thermal gravimetric analysis (TG-DSC) and Fourier-transform infrared spectroscopy (FTIR) characterization were performed. The thermal gravimetric results are presented in Figure 3a. The analysis revealed that the carbide slag sample undergoes three stages of weight loss during heating from 50 to 1000 °C, namely the loss of free water and gypsum dihydrate, the loss of calcium hydroxide, and the thermal decomposition of calcium carbonate. The primary decomposition of Ca(OH)_2_ occurs in the temperature range of 400–500 °C [37]. According to the results, calcium elements in carbide slag mainly exist in the form of Ca(OH)_2_, with a content of 82%, and the remaining small number of calcium elements exist in the form of CaO, CaCO_3_, and CaSO_4_ compounds. The thermal effect observed in the temperature range 900–1000 °C is attributed to the pyrolysis of calcium sulfate under a reduced nitrogen atmosphere [53]. The FTIR infrared analysis results in Figure 3b show that the carbide slag sample contains characteristic peaks of -OH, -SO_4_, -CO_3_, and free water, supporting the results of thermal gravimetric analysis. Observing the microstructure of carbide slag samples, the scanning electron microscopy (SEM) image in Figure 3c,d shows that the microstructure of carbide slag is mainly composed of Ca(OH)_2_ lamellar structure. Energy-dispersive X-ray spectroscopy (EDS) analysis of the surface element composition of the microstructure revealed that, in addition to the main elements Ca and O, there were also impurity elements, such as C, Al, and Si, on the microsurface.

### 3.2. Reaction Conditions for Ammonium Extraction

Calcium extraction from carbide slag can be achieved through two primary methods: acid extraction and ammonium salt extraction. The reaction principle in ammonium salt extraction is more complex and has been studied less than the acid-base neutralization reaction. In this experiment, in order to achieve better extraction efficiency, the reaction conditions for the extraction of utilizable calcium by ammonium acetate were explored. The study investigated the effect of ammonium acetate on the extraction efficiency of utilizable calcium under different conditions, including reaction temperature and time, liquid–solid ratio, and ammonium–calcium ratio.

**Figure 4:**
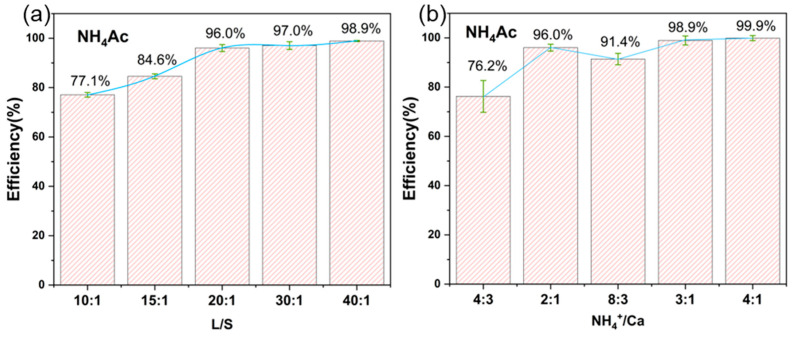
(**a**) Different liquid–solid ratios and (**b**) different ammonium–calcium ratios.

To investigate the effects of ammonium–calcium ratio and liquid–solid ratio on the efficiency of extractable calcium, the ammonium–calcium ratio was first fixed at the stoichiometric ratio (2:1), while the mass of the carbide slag sample remained constant. By adjusting the volume of ammonium acetate solution to change L/S, the Ca^2+^ concentration was measured after the carbide slag was reacted with the ammonium acetate solution for a sufficient amount of time at room temperature, and the extraction efficiency was calculated. As shown in Figure 4a, when L/S was low, so was the liquid–solid reaction efficiency, which resulted in the extraction efficiency being only 77%. When the liquid–solid ratio was increased to 20:1, the extraction rate rose to 96%, approaching complete reaction. Under the same reaction conditions, the ammonium acetate solution concentration was adjusted, and the extraction efficiency was calculated. As shown in Figure 4b, when the ammonium–calcium ratio was lower than the stoichiometric ratio, the reaction was incomplete, and the extractable calcium extraction efficiency was low. After the ammonium–calcium ratio reached 2:1, the extraction reaction was almost complete, and excess ammonium salt did not reduce the extraction efficiency. At an NH_4_/Ca ratio of 2:1 and an L/S ratio of 20:1, calcium extraction efficiency reached more than 90%. This rate surpasses the efficiency of other recent studies on the extraction of ammonium salt [49].

**Figure 5:**
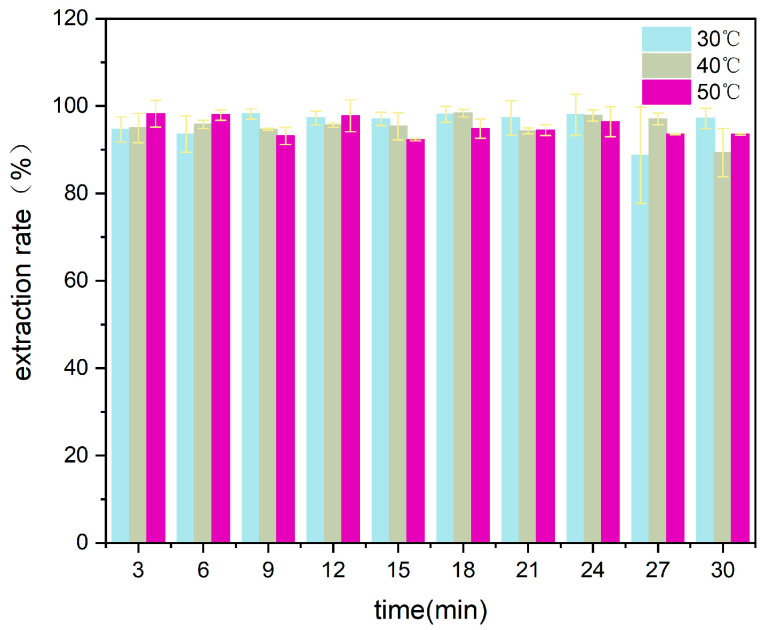
Effect of reaction time and temperature.

After determining the reaction conditions of L/S and NH_4_/Ca, the reaction temperature and time were experimentally explored. The system temperature was adjusted using an oil bath, and the Ca^2+^ concentration was measured every 3 min using a needle after sampling. As shown in Figure 5, the change in solution temperature had little effect on the extraction efficiency of Ca^2+^, and the extraction efficiency of calcium ions was above 90% at different temperatures. When the temperature was too high, ammonium hydroxide decomposed, which could easily lead to ammonia volatilization. The reaction time between carbide slag and ammonium acetate was short, and the reaction was completed within 5 min. Therefore, subsequent experiments were carried out under normal temperature and pressure conditions, and the reaction time was set to 5 min to save time and effort.

**Figure 6:**
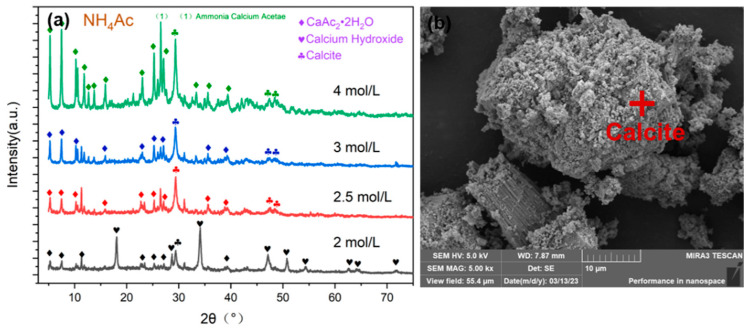
(**a**) XRD of different ammonium calcium ratio products and (**b**) microscopic electron microscopy of the residue.

To investigate the mineral composition of the residue after ammonium acetate extraction of utilizable calcium in carbide slag, X-ray diffraction (XRD) characterization was performed on the residue extracted using different concentrations of ammonium acetate solution. As shown in Figure 6a, when the concentration of ammonium acetate was low, the characteristic peak of Ca(OH)_2_ was present in XRD, indicating that the Ca(OH)_2_ in the carbide slag was not completely reacted. With the increase in ammonium acetate concentration, Ca(OH)_2_ was completely reacted, and precipitated calcium acetate crystals were observed in the residue due to the high concentration of calcium acetate in the solution. The residual residue after the extraction of utilizable calcium from the carbide slag mainly consisted of unreacted calcium carbonate in carbide slag, precipitated calcium acetate crystals, and other small amounts of insoluble impurities. Scanning electron microscopy (SEM) images (Figure 6b) showed that there was no Ca(OH)_2_ layer structure in the residue, indicating that most of the Ca(OH)_2_ in the carbide slag had participated in the reaction.

### 3.3. Comparison of Ammonium Extraction and Acid Extraction

**Figure 7:**
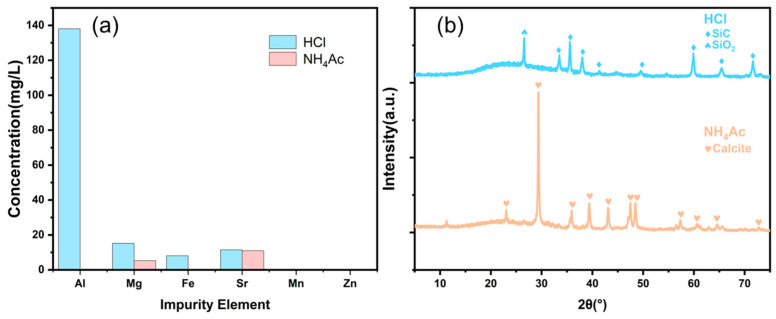
(**a**) Impurity concentration in solutions and (**b**) washed residue XRD.

Ammonium salt extraction has been found to have several advantages over acid extraction, including the absence of pH adjustment requirements and the recyclability of ammonium salt [54]. However, there has been limited research on the selective dissolution of available calcium by ammonium salts. After the ammonium salt solution reacts, the pH of the ammonium hydroxide solution exceeds 9, causing metal ions such as Al and Mg in the liquid phase to exist in the form of insoluble substances, which are then removed from the system by solid–liquid separation. Compared with acid extraction, ammonium salt extraction has the added advantage of high selectivity for impurity removal and can be used to prepare high-purity calcium carbonate. To verify the impurity removal effect of ammonium acetate extraction of utilizable calcium from the carbide slag, the multi-metal element concentrations of the reaction solutions after solid–liquid separation of acid extraction and ammonium salt extraction were measured by inductively coupled plasma (ICP). As shown in Figure 7a, the concentrations of Al and Mg impurities were higher in the reaction solution of acid extraction. In contrast, the metal ion impurities in the ammonium salt extraction solution were almost absent in the liquid phase system, indicating that the ammonium acetate solution had high selectivity for dissolving utilizable calcium in carbide slag.

As per the XRD characterization results presented in Figure 7b, the residue from the ammonium acetate extraction of utilizable calcium in carbide slag after washing was mainly composed of calcium carbonate in carbide slag that did not participate in the reaction. In contrast, the residue from hydrochloric acid extraction of utilizable calcium in carbide slag was a small amount of inert silicate compound. The amount of solid residue from acid extraction was smaller (1%), while the amount of residue from ammonium acetate extraction was greater (15%).

### 3.4. Effect of CO_2_ Mineralization

**Figure 8:**
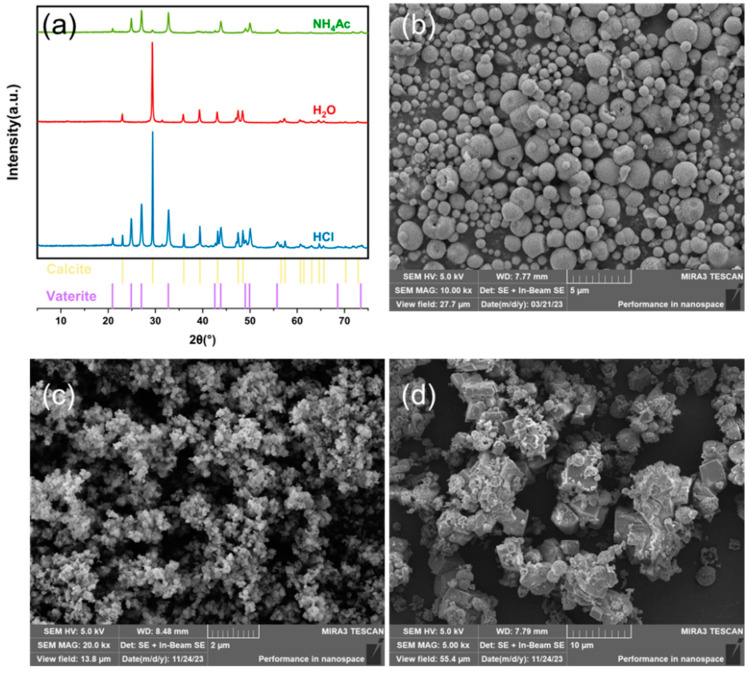
(**a**) CaCO_3_ XRD in different synthetic forms. (**b**) Ammonium acetate synthesis CaCO_3_ SEM. (**c**) Direct water carbonation CaCO_3_ SEM. (**d**) Acid extraction carbonization synthesis CaCO_3_ SEM.

After extracting calcium from calcium carbide slag with ammonium acetate solution, the pH of the liquid phase obtained by solid–liquid separation was measured to be 10, indicating an alkaline solution. Herein, pure CO_2_ was passed through the solution at a gas flow rate of 100 mL/min. After 15 min, the reaction solution was reduced to neutral. An amount of 5 g CaCO_3_ was prepared with 100 mL solution, which means the adsorption capacity of the solution was 22 g CO_2_/L. Nitrogen was used as equilibrium gas to simulate the flue gas CO_2_ concentration (15%). The reaction time was extended to 210 min due to the decreased concentration difference on the gas film side. The efficiency of carbon capture at low concentrations of CO_2_ can be improved by adding ammonia [37]. After centrifugation, the ammonium salt solution had the possibility of recycling, and the solid was characterized. The results are shown in Figure 8. XRD pattern analysis (Figure 8a) revealed that the crystal form of the CaCO_3_ product prepared by the ammonium acetate extraction process was vaterite crystal form, without impurity peaks. SEM Figure 8b showed that the microstructure of the CaCO_3_ products was entirely a spheraragonite structure, and there was no calcite crystal phase. The macrosphere was formed by the agglomeration of small spindle particles with an average particle size of less than 10 microns. With the same liquid–solid ratio, the crystal form of CaCO_3_, or calcite, prepared by direct carbonation of an aqueous suspension of calcium carbide slag is shown in Figure 8a,c. Microscopic images show that impurities are attached to the surface. XRD analysis of calcium carbonate synthesized by direct carbonation and ammonium salt are aligning with prior experimental findings [32,49].

To compare the difference between the extraction process of utilizable calcium ammonium salt and the extraction process of acid in the CO_2_ mineralization reaction, the solution after extraction of utilizable calcium by hydrochloric acid was added to ammonia, the pH was adjusted to 10, and CO_2_ was injected. The reaction conditions were the same as the mineralization reaction of ammonium acetate solution, and calcium carbonate products were successfully prepared after the pH was adjusted. The calcium carbonate products were characterized after solid–liquid separation and drying, and the results are shown in Figure 8a,c. XRD analysis revealed that the crystal form of CaCO_3_ products prepared by the hydrochloric acid extraction process comprised calcite crystal and vaterite in coexistence. SEM (Figure 8c) diagram showed that the CaCO_3_ had coexistent stable square structures and unstable spherical structures, and the particle size of CaCO_3_ prepared by acid solution was larger than that of nano-calcium carbonate prepared by ammonium acetate solution.

It is known that vaterite is a less thermodynamically stable form of CaCO_3_ than calcite. CaCO_3_ typically crystallizes via amorphous CaCO_3_ (ACC) phases that act as transient precursors [55]. The least stable vaterite can be stabilized in an aqueous solution at ambient conditions preventing its transformation into calcite [42]. In the ammonium synthesis system, vaterite was formed and stabilized. This was related to the impurity components (point defects) in the aqueous solution. In direct water carbonation, CaCO_3_ crystallized as the most thermodynamically stable calcite, which matched the results from the literature [30]. However, in the acid synthesis system, two crystal forms of vaterite and calcite appeared.

**Figure 9:**
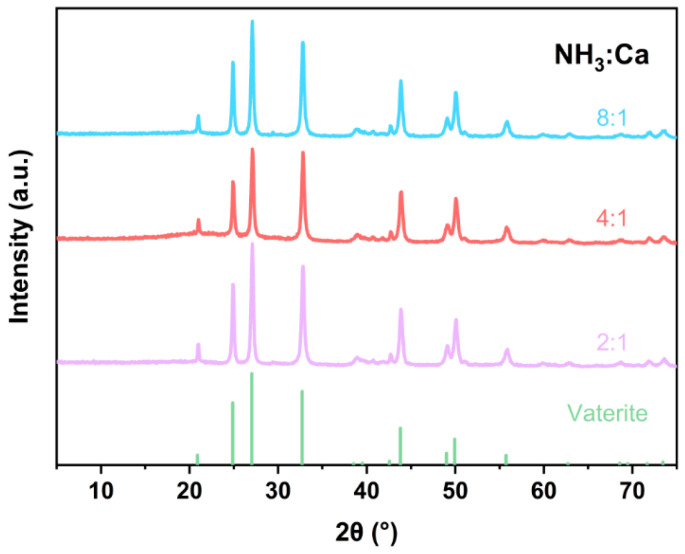
Different NH_3_:Ca ratios CaCO_3_ XRD.

To investigate the reasons for the different crystal types of CaCO_3_ prepared in two different reaction systems, we here discuss the different factors present in the reaction systems. In the acid synthesis system, the NH_3_/Ca ratio in solution changed due to pH-swing. A pure phase chemical reaction model was used to investigate the effect of ammonia concentration. The other reaction conditions were unchanged, and the amount of NH_4_^+^ and NH_4_OH in the solution was adjusted using NH_4_Ac and NH_4_OH as ammonia sources and CaCl_2_ as calcium sources. Mineralization reactions with different NH_3_/Ca ratios were constructed. CaCO_3_ prepared under different NH_3_/Ca ratios was characterized by XRD, as shown in Figure 9. It can be seen that under different NH_3_/Ca ratios, the CO_2_ mineralization products were all vaterite. This indicates that ammonia exists as an impurity in the aqueous solution, which makes ACC crystallize into vaterite and stabilize [56]. Therefore, ammonia concentration did not lead to the transformation of vaterite to calcite in this experimental condition.

**Figure 10:**
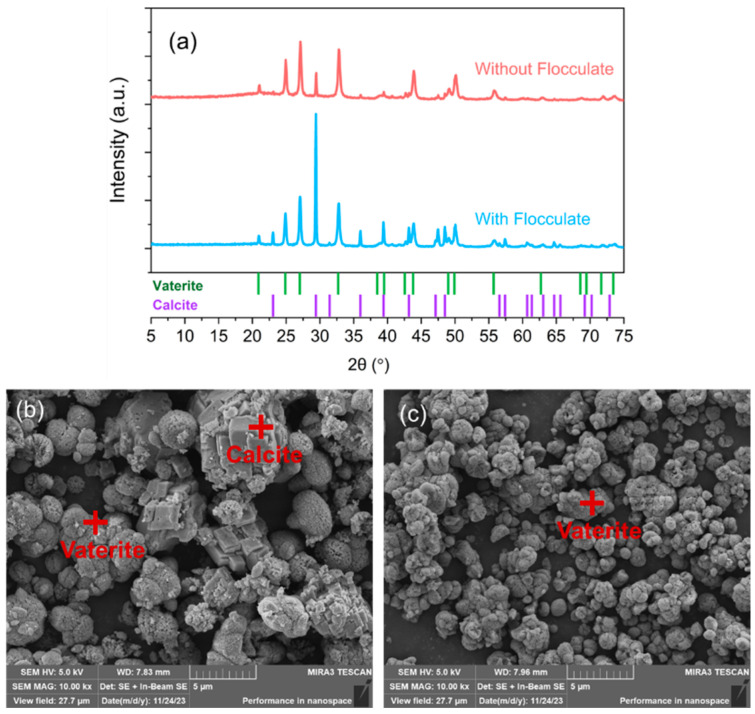
(**a**) The flocculation affects CaCO_3_ XRD; (**b**) SEM of CaCO_3_ with flocculation; (**c**) SEM of CaCO_3_ without flocculation.

It is reported that other ions (Al^3+^, Mg^2+^) influence the polymorphs of CaCO_3_ [57]. In the acid synthesis system, utilizable calcium extraction is accompanied by the dissolution of impurity ions (Figure 7a). After pH-swing, the impurity ions form a white gelatinous precipitate. During the crystallization of CaCO_3_, initially formed ACC particles transform into the least stable vaterite. According to Ostwald ripening, a redeposition of the dissolved vaterite occurs at the large crystal surfaces until they disappear. The most soluble vaterite undergoes dissolution and crystallization, finally forming the most stable calcite [58]. The precipitation of impurities may increase the rate of dissolution and recrystallization of vaterite, leading to the transformation to calcite. After filtering out most of the flocculates, the content and crystallinity of calcite in CaCO_3_ decreased (Figure 10). The researchers also believe that the presence of impurity ions in the alkaline environment will lead to calcite with higher roughness and surface imperfections [25].

## 4. Economic Analysis

According to the latest data, the annual production of calcium carbide slag in China is about 32.4 million tons, of which less than 40% were utilized [59]. There has been research that has established a relationship between the parameters and the CO_2_ emission life-cycle assessment, which, using preliminary economic analysis, suggests the profit from annual treatment of 1000 tons CO_2_ can reach more than 2100 CNY·(t CO_2_)^−1^ [37]. As calculated from the adsorption capacity of the solution obtained from the experiment herein, annual treatment of 1000 tons CO_2_ could use 2272 tons of calcium carbide slag. The transport expenses of raw materials can be addressed by integrating all chemical processes near the source of carbon emissions. According to the preliminary economic assessment, the main cost is the extractant consumption [25]. Therefore, the technology of regenerating the carbonized ammonium salt is being studied, as this technology will significantly reduce the cost. In the context of the development of China’s carbon trading market and the absence of carbon tax, more fiscal incentives can accelerate the realization of an effective zero-emission system [60]. In the future, we will optimize the integration process by applying Life Cycle Assessment (LCA) and Techno-Economic Assessment (TEA) to obtain a more sustainable and cost-effective process [61].

## 5. Conclusions

This paper defines calcium in carbide slag that can participate in liquid-phase carbonation reactions as utilizable calcium and explores the effects of different extraction processes on the efficiency of utilizable calcium extraction and CO_2_ mineralization. Compared with traditional direct water carbonation and acid extraction processes for carbide slag, ammonium salt extraction of utilizable calcium in carbide slag has several advantages, including high selectivity, high purity of calcium carbonate, and small particle size. The extracted ammonium water can be used to capture CO_2_ without pH adjustment. Additionally, ammonium salt recycling after carbonization is feasible and has high economic benefits. The prepared calcium carbonate product was a high-value-added vaterite crystal type.

This work explores the reaction conditions for ammonium acetate extraction of utilizable calcium in carbide slag, which can achieve an extraction efficiency of over 95% under normal temperature and pressure conditions with a liquid–solid ratio of 20:1 and an ammonium–calcium ratio of 2:1, while removing most of the impurity elements. The effects of two different extractants on CO_2_ mineralization were investigated. The calcium carbonate product prepared by ammonium acetate extraction of available calcium in carbide slag is a vaterite crystal type with low particle size and high purity. The product of calcium carbonate prepared by hydrochloric acid extraction of available calcium from calcium carbide slag is affected by impurity elements, and the product crystal form is affected by the coexistence of calcite and vaterite crystal. This article provides new perspectives and theoretical data for the high-value utilization of carbide slag.

## Figures and Tables

**Table 1:** Chemical components of the carbide slag.

Component	Values (%)
CaO	70.84
SiO_2_	1.89
Al_2_O_3_	1.06
SO_3_	0.306
Fe_2_O_3_	0.237
MgO	0.194
LOI	25.24

## Data Availability

Data are contained within the article.
